# CircRNA_000203 enhances the expression of fibrosis-associated genes by derepressing targets of miR-26b-5p, Col1a2 and CTGF, in cardiac fibroblasts

**DOI:** 10.1038/srep40342

**Published:** 2017-01-12

**Authors:** Chun-Mei Tang, Ming Zhang, Lei Huang, Zhi-qin Hu, Jie-Ning Zhu, Zhen Xiao, Zhuo Zhang, Qiu-xiong Lin, Xi-Long Zheng, Min -Yang, Shu-Lin Wu, Jian-Ding Cheng, Zhi-Xin Shan

**Affiliations:** 1 Guangdong Cardiovascular Institute, Guangdong Provincial Key Laboratory of Clinical Pharmacology, Guangzhou 510080, China; 2 Guangdong General Hospital, Guangdong Academy of Medical Sciences, Guangzhou 510080, China; 3 Southern Medical University, Guangzhou 510515, China; 4 Department of Forensic Pathology, Zhongshan School of Medicine, Sun Yat-sen University, Guangzhou 510080, China; 5 The Libin Cardiovascular Institute of Alberta, Department of Biochemistry & Molecular Biology, The University of Calgary, Calgary, T2N 1N4, Canada

## Abstract

Circular RNAs (circRNAs) participate in regulating gene expression in diverse biological and pathological processes. The present study aimed to investigate the mechanism underlying the modulation of circRNA_000203 on expressions of fibrosis-associated genes in cardiac fibroblasts. CircRNA_000203 was shown upregulated in the diabetic mouse myocardium and in Ang-II-induced mouse cardiac fibroblasts. Enforced-expression of circRNA_000203 could increase expressions of Col1a2, Col3a1 and α-SMA in mouse cardiac fibroblasts. RNA pull-down and RT-qPCR assay indicated that circRNA_000203 could specifically sponge miR-26b-5p. Dual luciferase reporter assay revealed that miR-26b-5p interacted with 3′UTRs of Col1a2 and CTGF, and circ_000203 could block the interactions of miR-26b-5p and 3′UTRs of Col1a2 and CTGF. Transfection of miR-26b-5p could post-transcriptionaly inhibit expressions of Col1a2 and CTGF, accompanied with the suppressions of Col3a1 and α-SMA in cardiac fibroblasts. Additionally, over-expression of circRNA_000203 could eliminate the anti-fibrosis effect of miR-26b-5p in cardiac fibroblasts. Together, our results reveal that suppressing the function of miR-26b-5p contributes to the pro-fibrosis effect of circRNA_000203 in cardiac fibroblasts.

Diabetes mellitus (DM) causes biochemical, functional and morphological abnormalities of cardiomyocytes. Individuals with DM are at a significantly greater risk of developing diabetic cardiomyopathy (DCM). DCM is characterized by structural and functional remodeling in the heart of diabetics even in the absence of coronary atherosclerosis, hypertension and other cardiac pathologies^1^,^2^,^3^,^4^,^5^. Hyperglycemia, insulin resistance, increased fatty acid metabolism, microcirculatory changes and myocardial fibrosis collectively contribute to the pathology of DCM^6^. A consistent association between the presence of cardiac hypertrophy and myocardial fibrosis and DCM has been revealed in previous studies^7^,^8^. Myocardial fibrosis enhances myocardial stiffness, decreases myocardial shortening and also provides the structural substrate for atrial and ventricular arrhythmias^9^. However, the mechanism involved in diabetic myocardial fibrosis remains to be elucidated.

MicroRNAs (miRNAs) are endogenous, 20–23 nucleotide RNAs that negatively regulate target genes involved in various physiological processes and diseases. For example, miR-29b^10^, miR-101^11^ and miR-132/-212^12^ were demonstrated implicated in myocardial fibrosis. However, circular RNAs (circRNAs) are a large class of endogenous non-coding RNAs, which form covalently closed continuous loops with high tissue-specific expression^13^. They mainly arise from exons or introns, and are differentially generated by back splicing or lariat introns^14^. Compared to linear RNAs, circRNAs have the remarkable characteristic of non-canonical splicing without a free 3′or 5′end^15^. CircRNAs could specifically function as microRNA (miRNA) sponges, regulate alternative splicing, and modulate the expression of parental genes^13^,^15^,^16^,^17^,^18^. Increasing evidence reveals that circRNAs may be involved in atherosclerotic vascular disease risk, neurological disorders, prion diseases and cancer^19^,^20^,^21^, holding great potential for novel clinical diagnostic and prognostic markers, as well as new treatment targets of diseases^22^,^23^,^24^.

In this study, circRNA_000203 was observed up-regulated in the diabetic mouse myocardium and in Ang-II-induced mouse cardiac fibroblasts. CircRNA_000203 was observed containing two potential binding sites for microRNA-26b-5p (miR-26b-5p), which exerted the anti-fibrotic effect by targeting Col1a2 and CTGF. Over-expression of circRNA_000203 could eliminate the anti-fibrotic effect of miR-26b-5p in cardiac fibroblasts. Our data suggest that blocking the function of miR-26b-5p contributes to the pro-fibrosis effect of circRNA_000203 in cardiac fibroblasts.

## Methods

### Ethics Statement

Sixteen-week-old male diabetic db/db mice and db/m control mice (Nanjing Biomedical Research Institute of Nanjing University, Nanjing, China) were housed under a 12-h light/dark cycle under pathogen-free conditions and fed standard mouse chow with free access to tap water. The 1- to 3-d old newborn C57BL/6 mice (License number SCXK (YUE) 2004–0011, Department of Experimental Animal Research Center, Sun Yat-sen University, Guangzhou, China) were used in the current studies. Mice were housed under a 12-h light/dark cycle under pathogen-free conditions and with free access to standard mouse chow and tap water. This study conformed to the Guide for the Care and Use of Laboratory Animals published by the US National Institutes of Health (8th Edition, National Research Council, 2011). All methods and experimental protocols in the present program were also approved by the research ethics committee of Guangdong General Hospital (the approval number: No. GDREC2012108A(R1)).

### Histological analysis

Mice were sacrificed by an intraperitoneal injection of 2 mL of pentobarbital. The left ventricular myocardium was fixed overnight in 10% formalin, followed with paraffin embedding and 4 μm thickness sectioning. They were mounted on normal glass slides and stained with Masson trichrome for histological examination. For the collagen volume fraction (CVF) analysis in the border zone of the infarcted region, eight separate views (magnification = original × 400) were selected and assessment of CVF used the following formula: CVF = collagen area/total area.

### Mouse cardiac fibroblasts (CFs) isolation, culture and treatment

Mouse cardiac fibroblasts (CFs) were isolated from 1–3 days old C57BL/6 mice by using a modification of previous report^25^. Briefly, CFs were separated from cardiomyocytes by gravity separation and grown to confluency on 10-cm cell culture dishes in growth media (DMEM/LG 10% FBS, 1% penicillin and 1% streptomycin) at 37 °C in humid air with 5% CO2. CFs from the third passage were used for experiments.

CFs were incubated with 10^−5^ M Ang-II for 24 h to induce the fibrotic phenotype. Cells were transfected with 50 nM scramble negative control (NC), miR-26b-5p mimic or miR-26b-5p-mut mimic (Ribobio, Guangzhou, China) by oligofectamine reagent (Invitrogen, Carlsbad, CA). As indicated, CFs were infected with the following recombinant adenovirus, respectively: rAd-GFP, rAd-CircRNA_000203 adenovirus (MOI 10).

### Circular RNAs microarray

CircRNAs expression analysis was performed on pooled total RNA extracted from myocardium of the 8 matched db/db mice and db/m control mice, respectively. Microarray procedures and data analysis were performed at Shanghai Kangchen Corporation. Briefly, total RNA was digested with Rnase R (Epicentre, Madison, WI, USA) to remove linear RNA and enrich circular RNA. Then, the enriched circular RNA was amplified and transcribed into fluorescent cRNA utilizing a random priming method (Arraystar Super RNA Labeling Kit; Arraystar). The labeled cRNAs were hybridized onto the Arraystar Human circRNA Array (8 × 15 K, Arraystar) (Rockville, MD, USA). After having washed the slides, the arrays were scanned by the Agilent Scanner G2505C. Agilent Feature Extraction software (version 11.0.1.1) was used to analyze acquired array images.

### Constructions of recombinant vectors for over-expression of circRNA_000203

Since circRNA_000203 cover the exon sequence from exon 7 to 15 of Myo9a gene, to generate the template DNA of circRNA_000203, a dsDNA with 1412 bp in length was synthysized, containing the exon sequence from exon 7 to 15, and the flanking sequence of intron 6 and intron 15 of Myo9a gene and the additional sequence of restriction enzyme *Xho*I and *Eco*RI at the two ends of this dsDNA (DNA sequence was shown in Supplementary Figure 1).

According to our previous report^26^, the template DNA of circRNA_000203 was directionally inserted into the multiple cloning site in the pDsRed2-N1 vector (Invitrogen, USA) and pAd-Track-cmv vector(Coloncancer, USA), respectively. Then, pAd-Track-cmv-circRNA_000203 was further used to construct the recombinant adenovirus plasmid with pAdEasy-I (Coloncancer, USA) plasmid in BJ5183 *E.Coli*. Finally, the recombinant circRNA_000203 adenovirus plasmid was linearized by restriction enzyme *Pme*I and transfected into 293 T cells for package of circRNA_000203 recombinant adenovirus. Meanwhile, the adenovirus rAd-GFP control vector was also prepared.

### Quantitative measurements of concerned genes mRNA, circRNA_000203 and miR-26b-5p

For detection of mRNA expression of coding genes, first-strand cDNAs were generated from 1.5 μg total RNA using a mixture of oligo (dT)_15_ and random primers with superscript reverse transcriptase (Invitrogen, Carlsbad, CA). RT-qPCR for miR-26b-5p was performed on cDNA generated from 0.5 μg total RNA according to the manufacturer’s protocol (Ribobio, Guangzhou, China). GAPDH was used for template normalization of coding genes and circRNA_000203, and U6 was used for miR-26b-5p template normalization. PCR and analyses were performed with a vii A7 Quantitative PCR System (Applied Biosystems, Carlsbad, CA). The 2-^∆∆Ct^ method was used to calculate relative expression levels of coding genes and miR-26b-5p. PCR primers, as well as the size of fragments amplified, used in this study are shown in Supplementary Table 1.

### RNA pull-down assay

pAd-Track-cmv-circRNA_000203 was transfected into HEK293 cells to overexpress circRNA_000203. Then the total RNA was extracted from the HEK293 cells with enforced expression of circRNA_000203, and 100 μg total RNA was used to incubated with 500 μg streptavidin magnetic beads (S1421S, NEB, Ipswich, MA, USA) which were previously incubated with 200 pmol biotin-miR-26b-5p or 200 pmol biotin-miR-26b-5p-mut (CAAG in the seed sequence of miR-26b-5p was altered by GUUC), respectively. Eventually, the binding RNA was eluted and used for circRNA_000203 detection by RT-qPCR assay.

### Dual luciferase assay for Col1a2 and CTGF targets identification

As in our previous report^27^, the recombinant luciferase reporter plasmids containing the potential miR-26b-5p binding site sequences of the Col1a2 and CTGF genes were constructed. Human embryonic kidney (HEK) 293 cells (3 × 105 cells per well in 12-well plate) were co-transfected with 200 ng of recombinant luciferase reporter plasmid, 20 ng of pRL-TK as an internal control (Promega, Madison, WI), 200 ng of pDsRed2-N1 or 200 ng of pDsRed2-circRNA_000203, 50 nM miR-26b mimic or 50 nM mutant miR-26b mimic (the seed sequence of miR-26b UCAAGUA was changed with UGUUCUA), respectively. Activities of firefly luciferase (FL) and Renilla luciferase (RL) were measured 24 hr after transfection, and the relative ratio of the FL/RL was used to indicate the miR-26b-mediated knockdown of target genes.

### Fluorescence Immunohistochemistry (FIHC)

Cultured CFs were washed in phosphate-buffered saline, fixed for 10 min in 3.7% formaldehyde, and permeabilized for 10 min in 0.1% Triton X-100. Cell monolayers were then washed in blocking solution and incubated with anti-Col1a2 antibody, anti-Col3a1 antibody and anti-α-SMA antibody (Santa Cruz Biotechnology, Santa Cruz, CA, USA) over night at 4 °C, respectively, followed by incubation with Alexa Fluor^®^ 555 donkey anti-rabbit IgG antibody (Molecular Probes, Eugene, OR, USA) for 1 h at room temperature. Confocal micrographs were obtained using a Leica SP5 confocal microscope (Leica, Mannheim, Germany). The fluorescence intensity analysis was performed using the LAS AFTCS SP5 imaging software.

### Western blot analysis

The amount of 40 μg protein prepared from mouse cardiac fibroblasts was used in a standard Western blot analysis. The polyvinylidene fluoride (PVDF) membrane binding sample protein was incubated with a high affinity anti-Col1a2 antibody (1:1000), anti-Col3a1 antibody (1:1000), anti-α-SMA (1:2000), anti-CTGF (1:2000) (Santa Cruz Biotechnology, Santa Cruz, CA, USA) overnight at 4 °C. Membranes were then washed extensively with TBS/T and incubated with a horseradish peroxidase (HRP)-conjugated secondary antibody (1:5000) (Santa Cruz Biotechnology, USA) for 1 h at room temperature. As an internal control, membranes were also immunoblotted with an anti-GAPDH antibody (1: 2000) (Santa Cruz Biotechnology, USA).

### Statistical analysis

The data are presented as the means ± s.e.m. In each experiment, all determinations were performed at least in triplicate. Statistical significance between two measurements was determined by the two tailed unpaired Student’s *t* test, and among groups, it was determined by one-way ANOVA. A value of *p* < 0.05 was considered to be significant.

## Results

### Upregulation of CircRNA_000203 in the diabetic mouse myocardium

**Figure 1:**
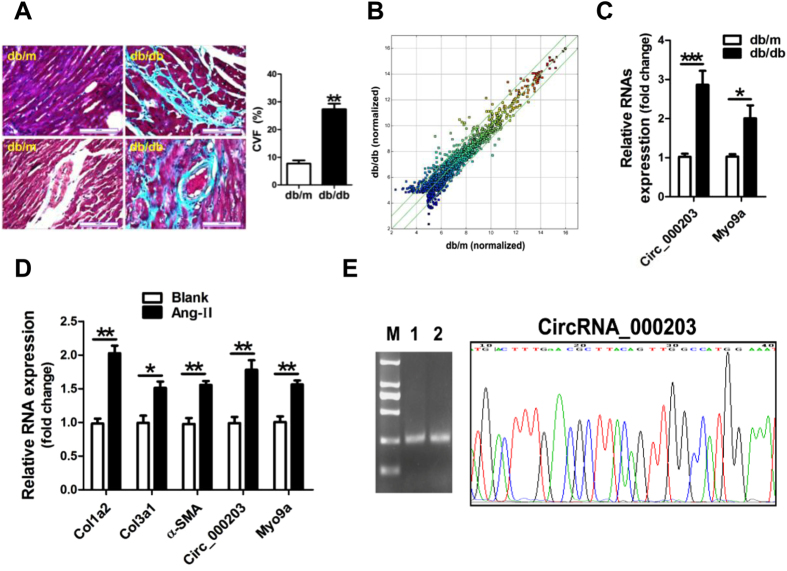
CircRNA_000203 expression in the diabetic mouse myocardium. (**A**) Masson trichrome staining. scale bar is 100 μm. (**B**) The Scatter Plot figure showed the representative dysregulated circRNAs in the diabetic mouse myocardium. The values of X and Y axes in the Scatter-Plot are the normalized signal values of the samples (log2 scaled). The green lines are fold change Lines. The circRNAs above the top green line and below the bottom green line indicated more than 2.0 fold change of circRNAs between the two compared samples. (**C**) Expressions of cirRNA_000203 and Myo9a mRNA in the diabetic mouse myocardium by RT-qPCR assay. (**D**) Expression of Col1a2, Col3a1, α-SMA, circ_000203 and Myo9a in Ang-II-induced mouse cardiac fibroblasts by RT-qPCR assay. (**E**) PCR product of circRNA_000203 was identified by 1.5% agarose gel electrophoresis and DNA sequencing assay, respectively. M, DL2000 DNA marker (from up to down, 2000, 1000, 750, 500, 250, 100 bp), lane 1, 2, PCR product of circRNA_000203. Data are shown as mean ± sem, *p < 0.05, **p < 0.01, ***p < 0.001. N = 5–8 in (**A**,**C**), and N = 3 in (**D**).

Results of Masson trichrome staining revealed that the perivascular and interstitial fibrosis was markedly increased in the diabetic mouse myocardium (Fig. 1A). A circRNAs-profiling array revealed that circRNAs were dysregulated in the diabetic mouse myocardium. Forty-five circRNAs were increased over 2-fold, meanwhile, thirty-one circRNAs were decreased more than 2-fold in the myocardium of the diabetic mice (Fig. 1B). Results of RT-qPCR verified that circRNA_000203 and its parental gene of Myo9a were significantly up-regulated in the myocardium of db/db mice compared to the db/m control mice (*p* < 0.05, *p* < 0.001, respectively) (Fig. 1C). A cell model of Ang-II-induced mouse cardiac fibroblasts was established, along with the significant increase of mRNA expression of Col1a2, Col3a1 and α-SMA (Fig. 1D). Consistently, circRNA_000203 and Myo9a were also observed markedly increased in Ang-II-treated cardiac fibroblasts (Fig. 1D). Gel electrophoresis result revealed that the size of PCR product of circRNA_000203 was the same as expected (Fig. 1E). In addition, DNA sequencing result confirmed that the sequence of PCR products of circRNA_000203 was correct (Fig. 1E).

### CircRNA_000203 enhances the expression of Col1a2, Col3a1 and α-SMA in mouse cardiac fibroblasts

**Figure 2:**
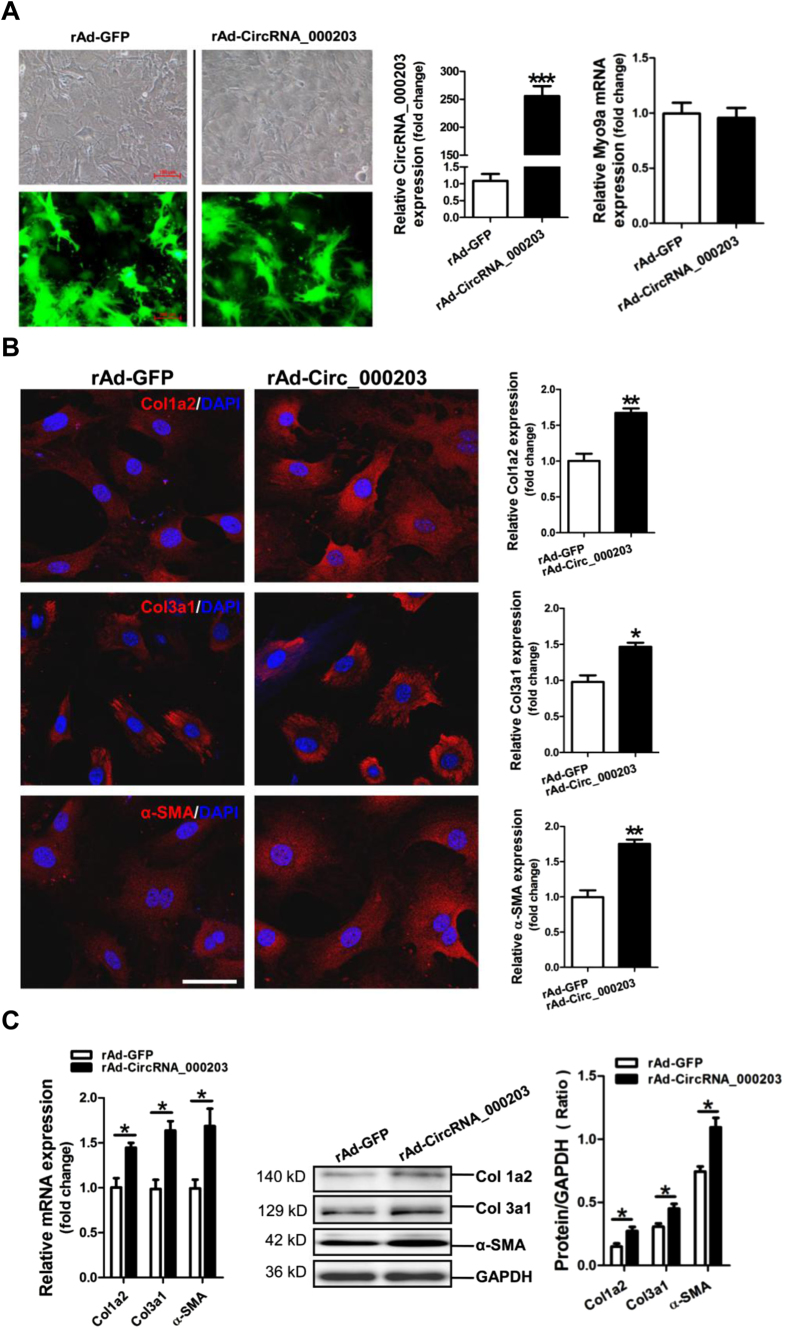
Effect of circRNA_000203 on expressions of Col1a2, Col3a1 and α-SMA in mouse cardiac fibroblasts. (**A**) Adenovirus-mediated overexpression of circRNA_000203 in cardiac fibroblasts. Infection of recombinant circRNA_000203 adenovirus was monitored by co-expressed marker of GFP. The scale bar is 100 μm. Expression of circRNA_000203 was determined by RT-qPCR assay. (**B**) Expressions of Col1a1, Col3a1 and α-SMA in cardiac fibroblasts with overexpression of circRNA_000203 by FIHC assay. The scale bar is 100 μm. (**C**) MRNA expression of Col1a2, Col3a1 and α-SMA in cardiac fibroblasts by RT-qPCR assay. (**D**) Protein expression of Col1a2, Col3a1 and α-SMA in cardiac fibroblasts by Western-blot assay. Data are shown as mean ± sem, *p < 0.05, **p < 0.01, ***p < 0.001. N = 3.

In this study, we constructed the recombinant circRNA_000203 adenovirus with the co-expression of GFP marker gene. GFP was observed highly expressed in CFs, which indicated that CFs were efficiently infected by the recombinant circRNA_000203 adenovirus. As expected, the RT-qPCR result showed that circRNA_000203 was dramatically upregulated in CFs infected with rAd-Circ_000203 (*p* < 0.001), without significant change of Myo9a mRNA (Fig. 2A). Results of FIHC assay revealed that Col1a2, Col3a1 and α-SMA expression was consistently enhanced by circRNA_000203 in CFs (*p* < 0.05, *p* < 0.01, respectively) (Fig. 2B). Additionally, the RT-qPCR and Western blotting assay demonstrated that circRNA_000203 could specifically increases the expression of Col1a2, Col3a1 and α-SMA in CFs (*p* < 0.05, respectively) (Fig. 2C).

### CircRNA_000203 abolishes the interaction of miR-26b-5p with the targets of Col1a2 and CTGF

**Figure 3:**
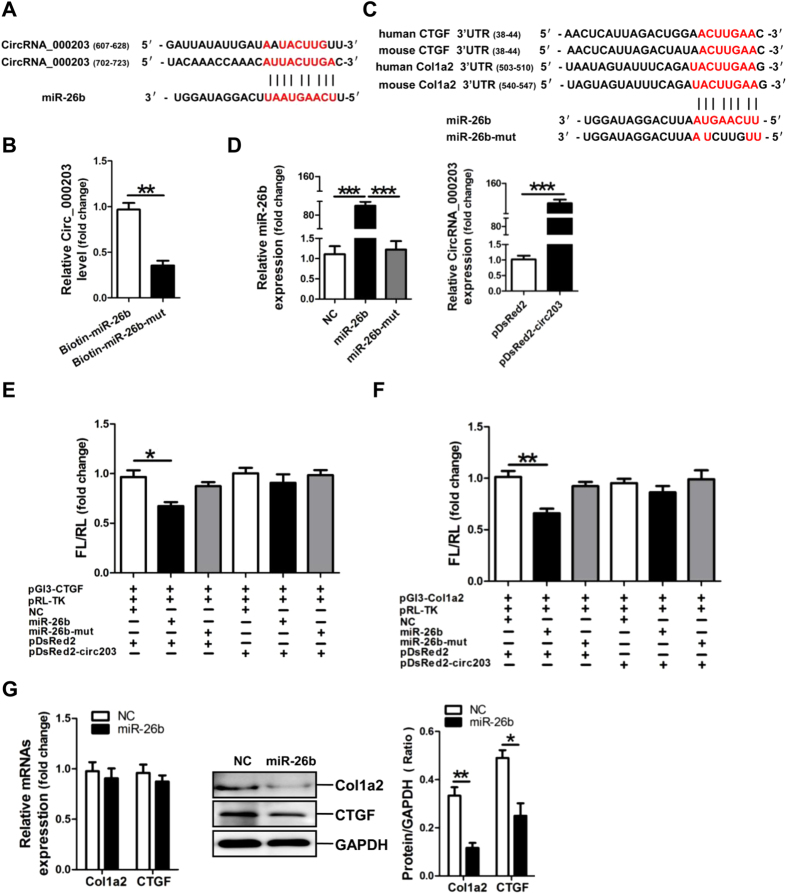
Identification of miR-26b as a target of circRNA_000203. (**A**) Predicted binding sites of miR-26b in circRNA_000203. (**B**) The pulled down circRNA_000203 by miR-26b or miR-26b-mut by RT-qPCR assay. (**C**) Predicted miR-26b seed matches to the sequences in the 3′UTR of CTGF and Col1a2 mRNA. The seed sequence of miR-26b is UCAAGUA, and the complementary nucleotide sequences are shown in red words. (**D**) Expressions of miR-26b and circRNA_000203 were determined by RT-qPCR assay. Verification of CTGF (**E**) and Col1a2 (**F**) as target genes of miR-26b by the dual luciferase reporter assay. (**G**) MRNA and protein expression of Col1a2 and CTGF in mouse cardiac fibroblasts with overexpression of miR-26b. Data are shown as mean ± sem, *p < 0.05, **p < 0.01, ***p < 0.001. N = 3.

Sequence analysis indicated that there are two potential binding sites, 607–628 nt and 702–723 nt, for miR-26b-5p in circRNA_000203 (Fig. 3A). The RNA pull-down and RT-qPCR assay showed that the amount of circRNA_000203 pulled down by miR-26b-5p-mut was significantly less than that by miR-26b-5p (Fig. 3B). Analysis of the databases Mirdb (www.mirdb.org) and TargetScan-Vert (www.targetscan.org) showed that Col1a2 and CTGF were potential target genes of miR-26b. The matching positions for miR-26b within 3′-UTR of the targeted mRNAs are shown in Fig. 3C. Results of RT-qPCR assay revealed that miR-26b mimic was efficiently delivered and circRNA_000203 was also highly expressed in HEK293 cells for the dual luciferase assay (Fig. 3D). The dual luciferase assay indicated that miR-26b mimic, but not miR-26b-mut, can specifically interact with the 3′-UTRs of CTGF (Fig. 3E) and Col1a2 gene (Fig. 3F), respectively. However, the interactions of miR-26b with the 3′-UTRs of CTGF and Col1a2 gene could be blocked by circRNA_000203 (Fig. 3E,F). Compared with the scramble negative control (NC), the expression Col1a2 and CTGF was significantly reduced at the protein level, but not at the mRNA level, in miR-26b mimic-modified mouse CFs (*p* < 0.05, *p* < 0.01, respectively) (Fig. 3G).

### CircRNA_000203 eliminates the antifibrotic effect of miR-26b in mouse CFs

**Figure 4:**
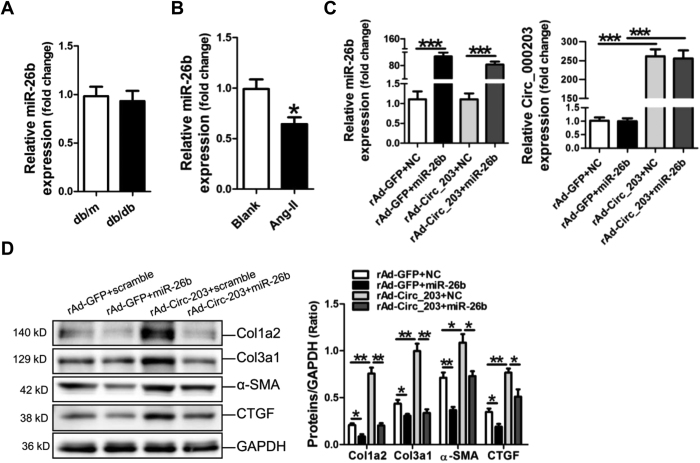
CircRNA_000203 reverses the inhibitory effect of miR-26b on expressions of fibrosis-associated genes. Expression of miR-26b in the diabetic mouse myocardium (**A**) and in Ang-II-induced mouse cardiac fibroblasts (**B**) by RT-qPCR assay. (**C**) Determinations of miR-26b and circRNA_000203 in cardiac fibroblasts by RT-qPCR assay. (**D**) Col1a2, Col3a1, α-SMA and CTGF protein expression in cardiac fibroblasts by Western blot assay. Data are shown as mean ± sem, *p < 0.05, **p < 0.01, ***p < 0.001. N = 3.

RT-qPCR assay showed that no significant change of miR-26b was found in the diabetic mouse myocardium (Fig. 4A), but miR-26b was obviously decreased in Ang-II-induced mouse CFs (*p* < 0.05) (Fig. 4B). To investigate the effect of circRNA_000203 on miR-26b-mediated suppression of fibrosis-associated genes, mouse CFs were infected with the recombinant circRNA_000203 adenovirus, followed with the transfection of miR-26b mimic and the scramble control, respectively. RT-qPCR results revealed that miR-26b mimic was efficiently delivered and circRNA_000203 was significantly upregulated in mouse CFs (Fig. 4C). The results of Western-blot demonstrated that the expression of fibrosis-associated genes, including Col1a2, Col3a1, α-SMA and CTGF, were significantly suppressed by miR-26b in moue CFs, however, circRNA_000203 could dramatically promote the expressions of the above genes, and eliminated the antifibrotic effect of miR-26b (Fig. 4D).

## Disccusion

Recently, thousands of circRNAs in the mammalian transcriptome have been discovered based on bioinformatics and experimental analysis^28^,^29^. Circular RNAs are characterized by the presence of a covalent bond linking the 3′ and 5′ ends generated by backsplicing^14^. Functionally, circRNAs have been found to act as microRNA (miRNA) sponges, regulators of alternative splicing, and even to serve as transcription factors to modulate the expressions of target genes^30^.

CircRNAs have been found involved in a serious of diseases^19^,^20^,^21^. In this study, dysregulation of circRNAs was observed in the diabetic mouse myocardium, indicating that circRNAs may participate in the process of DCM. Therefore, functional study of the dysregulated circRNAs will contribute to reveal the roles of circRNAs in DCM.

CircRNAs can be produced through back splicing of a precursor mRNA (pre-mRNA), and the spliceosome has been demonstrated involved in the exon circularization process^19^,^31^,^32^. The flanking intronic regions are complementary to each other, which is necessary for circularization of some circRNAs. For example, in human, these complementary sequences often consist of inverted Alu repeats^33^,^34^. In this study, a double strand template DNA, containing the exon sequence from exon 7 to 15, and the flanking sequence in intron 6 and intron 15 of Myo9a gene, was synthysized and cloned into pDsRed2-N1 plasmid vector and pAdTrack-cmv adenovirus vector, respectively. As expected, RT-qPCR result revealed that circRNA_000203 was efficiently over-expressed via plasmid vector in HEK293 cells and via adenovirus vector in cardiac fibroblasts. Our present data also indicated that circRNA_000203 was upregulated in consistent with its host gene Myo9a in the diabetic mouse myocardium and Ang-II-induced mouse cardiac fibroblasts, respectively.

The present study demonstrated that circRNA_000203 specifically enhances the expression of Col1a2, Col3a1 and α-SMA in cardiac fibroblasts, with no effect on the expression of the host gene Myo9a. Mechanically, two binding sites of miR-26b-5p in circRNA_000203 contributed to the sponge effect of circRNA_000203 on miR-26b-5p. Moreover, miR-26b-5p was verified to inhibit the fibrotic phenotype by suppressing the target genes of Col1a2 and CTGF in cardiac fibroblasts.

Our current study has provided several lines of evidence to support the notion that miR-26b-5p inhibits cardiac fibrosis through targeting Col1a2 and CTGF. First, the *in silico* prediction indicated that Col1a2 and CTGF were two potential targets of miR-26b-5p, and the dual luciferase assay revealed that miR-26b-5p specifically bound to the 3′-UTRs of Col1a2 and CTGF, respectively. Additionally, miR-26b-5p mimic inhibited Col1a2 and CTGF expression at the post-transcriptional level in cardiac fibroblasts. The present conclusion has been partially supported by previous studies showing that CTGF is a target of miR-26b in osteosarcoma cells^35^ and in pulmonary artery smooth muscle cells^36^, respectively. Importantly, our present data validated that circRNA_000203 specifically eliminates the interactions of miR-26b-5p with the 3′-UTRs of Col1a2 and CTGF, and impairs the inhibitory effect of miR-26b-5p on the fibrosis-associated genes, including Col1a2, Col3a1, α-SMA and CTGF, in cardiac fibroblasts.

**Figure 5:**
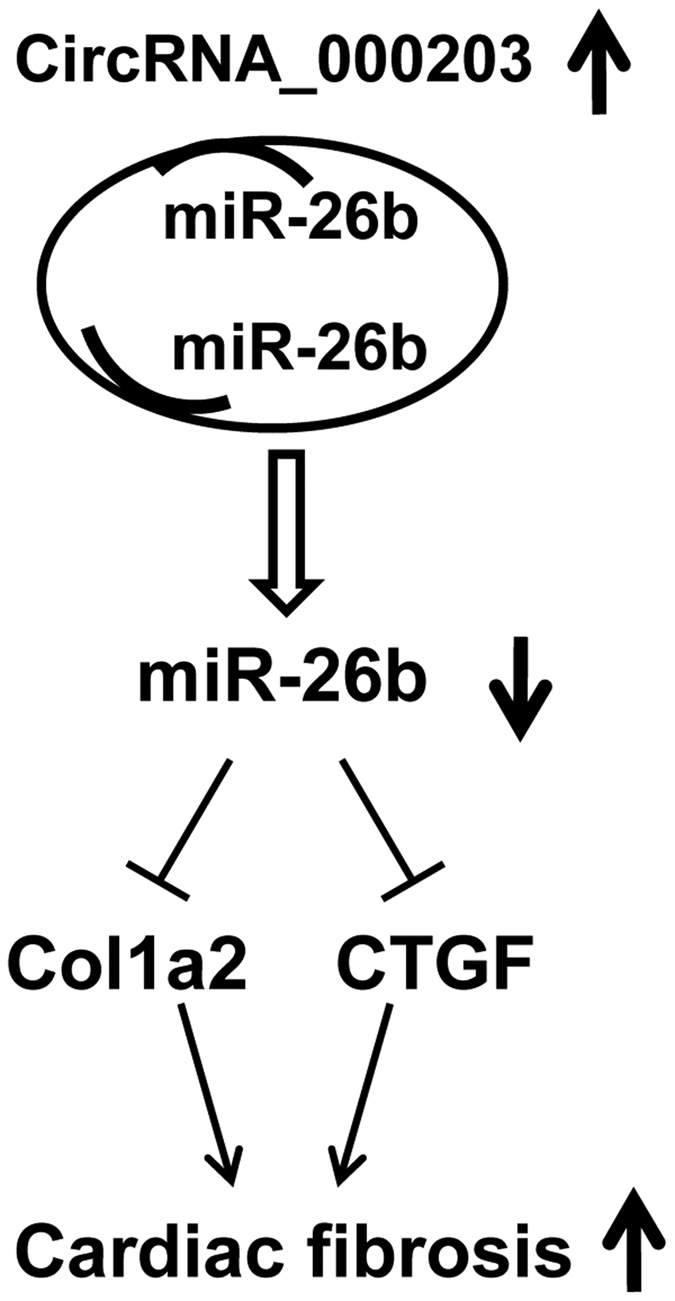
Schematic diagram of the mechanism whereby circRNA_000203 increases cardiac fibrosis. The binding interaction between circRNA_000203 and miR-26b derepresses the inhibitory effect of miR-26b on Col1a2 and CTGF in cardiac fibroblasts, resulting in the upregulation of Col1a2 and CTGF, and contributing to the enhancement of cardiac fibrosis.

Taken together, our results have revealed that circRNA_000203 was up-regulated in the diabetic myocardium and in Ang-II-induced cardiac fibroblasts. Functionally, circRNA_000203 could enhance the expressions of Col1a2, Col3a1 and α-SMA in cardiac fibroblasts. Mechanically, circRNA_000203 sponged miR-26b-5p to derepress the downstream targets of Col1a2 and CTGF, contributing to the enhancement of the fibrotic phenotype in cardiac fibroblasts (as shown in Fig. 5). Therefore, the present study suggests that circRNA_000203 might be a potential target for prevention and treatment of cardiac fibrosis in DCM.

## Additional Information

**How to cite this article**: Tang, C.-M. *et al*. CircRNA_000203 enhances the expression of fibrosis-associated genes by derepressing targets of miR-26b-5p, Col1a2 and CTGF, in cardiac fibroblasts. *Sci. Rep.* **7**, 40342; doi: 10.1038/srep40342 (2017).

**Publisher's note:** Springer Nature remains neutral with regard to jurisdictional claims in published maps and institutional affiliations.

## Supplementary Material

Supplementary Information
