# Supplementary material for: CircRNA_000203 enhances the expression of fibrosis-associated genes by derepressing targets of miR-26b-5p, Col1a2 and CTGF, in cardiac fibroblasts
Source: Sci Rep. 2017 Jan 12;7:40342. doi: 10.1038/srep40342 (PMC5228128; doi:10.1038/srep40342)
Supplement: Supplementary Information [file srep40342-s1.pdf]

CircRNA_000203 enhances the expression of fibrosis-associated genes by derepressing targets of miR-26b-5p, Col1a2 and CTGF, in cardiac fibroblasts

Chun-Mei Tang[1,2,3]* , Ming Zhang[2,]*, Lei Huang[4]*, Zhi-qin Hu[1,2,3]*, Jie-Ning Zhu[1,2], Zhen Xiao[2], Zhuo Zhang[2], Qiu-xiong Lin[1,2], Xi-Long Zheng[5], Min-Yang[1,2], Shu-Lin Wu[1,2], Jian-Ding Cheng[a,4] and Zhi-Xin Shan[a,1,2]

[1] Guangdong Cardiovascular Institute, Guangdong Provincial Key Laboratory of Clinical Pharmacology, Guangzhou 510080, China

[2] Guangdong General Hospital, Guangdong Academy of Medical Sciences, Guangzhou 510080, China

[3] Southern Medical University, Guangzhou 510515, China

[4] The Libin Cardiovascular Institute of Alberta, Department of Biochemistry & Molecular Biology, The University of Calgary, Calgary, T2N 1N4, Canada

[5] Department of Forensic, Sun-Yat-sen University, Guangzhou 510275, China

[a]Email: chengjd@mail.sysu.edu.cn

[a]Email: zhixinshan@aliyun.com

[*] These authors contributed equally to this work.

Supplementary table 1 Primers used in real-time qRT-PCR

Gene	Sequence (5′- 3′)	Product size (bp)
Col1a2	F, CTGGTCCTGTTGGAAGTCGT	201
	R, CAGATGCACCTGTTTCTCCA	
Col3a1	F, CAATGTAAAGAAGTCTCTGAAG	240
	R, CAAACAGGGCCAATGTCCAC	
α-SMA	F, CTGTGCTATGTCGCTCTGGA	192
	R, ATAGGTGGTTTCGTGGATGC	
CTGF	F,GAAGCTGACCTGGAGGAAAA	182
	R, ACTGGCAGAGTGGTGGTTCT	
Myo9a	F, TTTCTGGGATGACTGGCATT	191
	R, GACTGGGTGTTGGAGAAAGC	
circRNA_000203	F, AAGAGAAGTACAGATTGCTTCA	249
	R, CTCTTCTTTAACTTCTAATAATTC	
GAPDH	F, CAAGAAGGTGGTGAAGCAGG	200
	R, CCACCCTGTTGCTGTAGCC	
miR-26b	RT, GTCGTATCCAGTGCGTGTCGTGGAGTCGG CAATTGCACTGGATACGACACCTATCC	76
	F, TTCAAGTAATTCAGGATAGGT	
	R, GTGCGTGTCGTGGAGTC	
U6	RT, GTCGTATCCAGTGCGTGTCGTGGAGT CGGCAATTGCACTGGATACGAC	160
	F, GTCCGCGTGCTCGCTTCGGCAGC	
	R, GTGCGTGTCGTGGAGTC	

Supplementary figure 1. Template DNA sequence of circRNA_000203.

```
   1 gtcagattatatttaaatattctgttttactttgcatttacacagggccctacttaaatt
  61 aatgggtggcttacattgtttttaacaagtaaatgtgatatatattttcttccacctctt
 121 gtcttaagcaggattgcttcacagtggagggagaagatttgaggcatgactttgaacgct
 181 tacagttggccatggaaatggtaggatttcttcccaagacacgaagacagattttctctc
 241 tgctctcagcaatactacatttgggtaatatctcatataaaaagaagacctatcgggatg
 301 actctattgatatctgtaatcctgaagtactacccattgtctcagaattattagaagtta
 361 aagaagagatgctatttgaagcattagttacaaggaagacggtgacagtgggagaaaagc
 421 ttattttaccatataaactggcagaggctgtgacagtgagaaactccatggctaagtctt
 481 tatatagtgctctgtttgactggatagtgtttcgtattaatcatgcacttctgaatagta
 541 aagatttggaacaagatactaagactttgtccattggtgttcttgatatttttgggtttg
 601 aagattatgaaaacaatagttttgaacagttctgtattaattttgctaatgaacgtttac
 661 agcactactttaatcagcatatctttaagttggagcaagaggaatatagaactgaaggta
 721 tcagttggcataacatagattatattgataatacttgttgcataaatcttattagcaaaa
 781 aaccaacaggactgctccatcttttggatgaagaaagcaactttccacaggctacaaacc
 841 aaacattacttgacaagtttcaacatcaacatgaagagaattcttacattgaatttcctg
 901 ctgtgatggagcctgcttttatcataaaacattatgctggaaaagtaaaatatggagtaa
 961 aggattttcgggaaaaaaatacagatcatatgcgtccagacattgtggctcttctgagaa
1021 gtagcaggaatgcttttgtttctgggatgactggcattgatcctgtagctgttttccgat
1081 gggcagttcttcgagcatttttcagagctgtggttgctttcagggaagctgggaaaagac
1141 atattcagagaaaaagtggacatgatgatacaacaccatgtgcgattttgaaaagtatgg
1201 atagttttagctttctccaacacccagtccaccagaggagcttagagattctgcagaggt
1261 gcaaggaagagaagtacagtaaggctttaagtcctcttaaccctgtattgtcacatctat
1321 ttggaaatggaaatcaagagtattcatgagggatgcatggagattgcataggaacatgcc
1381 tttcacaattctttatgcagactgtattgctt  1412
```
